# Bias Characterization of Resting Energy Expenditure Prediction Equations in Japanese Subjects: A Pilot Exploratory Study

**DOI:** 10.3390/nu18162743

**Published:** 2026-08-21

**Authors:** Hitomi Matsuura, Kanako Deguchi, Katsumi Iizuka

**Affiliations:** 1 Department of Clinical Nutrition, School of Medicine, Fujita Health University, Toyoake 470-1192, Japan; 51023103@fujita-hu.ac.jp (H.M.); kanasakuran@gmail.com (K.D.); 2 Faculty of Medicine, Fujita Health University, Toyoake 470-1192, Japan

**Keywords:** Harris–Benedict equation, Ganpule equation, Cunningham equation, Dietary Reference Intakes for Japanese, portable indirect calorimetry, Bland–Altman analysis

## Abstract

Background: Accurate estimation of energy requirements is essential for appropriate nutritional therapy. Resting energy expenditure (REE) predictive equations are derived from specific populations and may therefore contain systematic individual-level biases when applied in different clinical settings. In this pilot exploratory study, we compared REE measured by portable indirect calorimetry with estimates obtained using multiple predictive methods in Japanese adults and characterized their association, absolute agreement, and error structure. Methods: Thirty-six university staff members and students were enrolled. The association and absolute agreement between REE measured by portable indirect calorimetry and estimates obtained using four predictive methods (Harris–Benedict, DRIs, Cunningham, and Ganpule) were evaluated using Spearman’s rank correlation coefficients, ICC(2,1), and Bland–Altman analyses. For equations demonstrating significant proportional bias, multivariate linear regression analyses were additionally performed using residual estimation error, calculated as (predicted REE-measured REE)/measured REE × 100, as the dependent variable. Results: Participants included 16 men and 20 women, with a mean age of 28.9 ± 10.3 years and BMI of 22.1 ± 3.1 kg/m2. Spearman’s rho ranged from 0.750 to 0.800, and ICC(2,1) estimates ranged from 0.736 to 0.766. Mean biases (predicted minus measured REE) were 50.7 kcal/day (95% CI, −20.1 to 121.4) for Harris–Benedict, −73.1 kcal/day (95% CI, −148.8 to 2.6) for DRIs, −94.7 kcal/day (95% CI, −161.7 to −27.8) for Cunningham, and −90.6 kcal/day (95% CI, −161.1 to −20.2) for Ganpule. The corresponding proportions of predictions within ±10% of measured REE were 41.7% (95% CI, 27.1–57.8%), 50.0% (95% CI, 34.5–65.5%), 55.6% (95% CI, 39.6–70.5%), and 52.8% (95% CI, 37.0–68.0%), respectively. Multivariate analyses suggested greater underestimation in females and a shift toward overestimation with higher BMI. Conclusions: Despite moderate-to-strong rank associations with measured REE, Bland–Altman analysis revealed proportional bias in three of the four predictive methods that was not apparent from the correlation coefficients alone. Exploratory analyses suggested that sex and BMI should be considered when interpreting the direction of prediction error. Because the precision of the limits of agreement was restricted by the small sample size, larger studies are needed to estimate individual-level agreement more precisely and to examine these error patterns in older adults and individuals with acute or chronic diseases.

## 1. Introduction

Assessment of energy requirements is routinely performed in nutritional therapy, and determining appropriate energy provision is clinically important [1,2]. Total energy requirements are generally estimated by multiplying resting energy expenditure (REE) by activity and stress factors. Conventional indirect calorimetry measuring both oxygen consumption and carbon dioxide production is regarded as the reference method for measuring REE; however, it is not feasible for all patients in routine clinical practice. Therefore, predictive equations are widely used in daily nutritional management [3,4].

In Japan, the Harris–Benedict, Cunningham, and Ganpule equations and methods based on the Dietary Reference Intakes for Japanese are commonly used [5,6,7,8,9,10]. The Harris–Benedict equation, originally reported in 1919, was developed from 239 healthy individuals, predominantly from a White Western population, using body weight, height, age, and sex as predictors [5]. The equation based on fat-free mass, often referred to in clinical practice as the Katch–McArdle formula, was proposed by Cunningham and estimates REE primarily from fat-free mass [6,7]. The method based on the Dietary Reference Intakes for Japanese estimates BMR by multiplying sex- and age-specific basal metabolic reference values by body weight [8]. The Ganpule equation was developed from data obtained in Japanese adults and incorporates body weight, height, age, and sex [9]. These methods differ in their source populations, historical periods, predictor variables, and metabolic measurement protocols.

Prediction accuracy may depend on the characteristics of the populations from which the equations were derived, and substantial individual-level estimation errors have been reported [11,12,13,14,15,16]. Frankenfield et al. emphasized that prediction equations should be generalized cautiously across age and ethnic groups because their accuracy may vary according to population characteristics [11]. Previous validation studies have frequently examined older adults, individuals with extreme BMI values, or patients with specific diseases. Although such studies are clinically important, prediction error in these populations may partly reflect extrapolation beyond the characteristics represented in the data underlying the equations. It therefore remains relevant to characterize individual-level error when equations are applied under relatively non-extreme conditions with respect to age and body size. Few studies have compared multiple commonly used equations with portable indirect calorimetry in Japanese adults while examining not only group-level agreement but also individual-level variability, proportional bias, and equation-specific error patterns.

Rather than developing a new prediction equation, the present pilot exploratory study examined four commonly used methods in 36 Japanese adults whose age and anthropometric characteristics broadly overlapped with the ranges represented in the source populations or data underlying the equations. We evaluated the association and absolute agreement between equation-derived estimates and REE measured by portable indirect calorimetry and characterized mean bias, individual-level variability, and proportional bias. For equations showing proportional bias, exploratory multivariable analyses were additionally conducted to identify participant characteristics associated with relative prediction error. The study aimed to provide practical information for interpreting the limitations and equation-specific error patterns of commonly used energy-expenditure estimates in clinical practice.

## 2. Materials and Methods

### 2.1. Participants

This study enrolled 36 university staff members and students aged 20–60 years at Fujita Health University. Participants were recruited using a written study recruitment notice. The convenience sample consisted of 15 Fujita Health University staff members (41.7%) and 21 undergraduate students (58.3%); no patients or outpatient clinic visitors were included. The study population was a convenience sample of eligible individuals who agreed to participate. Exclusion criteria included the use of low-dose oral contraceptives, evident menstrual disorders, pregnancy, and any other condition considered by the investigators to make participation inappropriate. The study population did not include participants with acute infectious illness or known chronic respiratory disease, including chronic obstructive pulmonary disease. No participant was excluded under the general discretionary criterion.

No conventional power calculation was performed because this pilot exploratory study was designed to estimate mean bias, limits of agreement, proportional bias, and equation-specific error patterns rather than to test a prespecified hypothesis of equivalence or interchangeability. The proportion of predictions within ±10% of measured REE was used as a descriptive measure of individual-level accuracy and not as a prespecified criterion for establishing clinical equivalence. Accordingly, the precision of the agreement estimates was evaluated using confidence intervals, and the limits of agreement were interpreted descriptively. All participants received written and oral explanations of the study and provided written informed consent. All 36 enrolled participants completed the required measurements and were included in the final analysis. The study was conducted in accordance with the Declaration of Helsinki and was approved by the Research Ethics Committee of Fujita Health University (approval no. HM25-305; approved 14 October 2025).

### 2.2. Measurement of REE

Measurements were conducted at approximately 11:00 a.m. in a room with an ambient temperature of 20.7–25.7 °C (mean ± SD, 23.7 ± 1.4 °C) and relative humidity of 21–57% (33.3 ± 11.1%). All participants were scheduled within approximately the same time window, although measurements were not necessarily initiated at exactly the same clock time. It was confirmed that at least 4 h had elapsed since the participant’s last meal.

Body weight, body fat percentage, and whole-body skeletal muscle mass were obtained using bioelectrical impedance analysis with the InBody Dial H20 (InBody Co., Ltd., Seoul, Republic of Korea). These body-composition variables were regarded as device-derived estimates rather than direct measurements. Participants were instructed to avoid strenuous exercise and dehydration before BIA measurement. Measurements were performed after bladder emptying and after approximately 5 min in the standing position. Participants were barefoot and stood on the foot electrodes while holding the hand electrodes according to the manufacturer’s standard procedure. A single measurement was obtained at approximately 11:00 a.m. after at least 4 h without food intake. Fat mass was calculated as body weight × body fat percentage/100, and fat-free mass was calculated as body weight − fat mass. Raw resistance and reactance values were not recorded or exported and were unavailable for analysis. Skeletal muscle mass index (SMI) was calculated as whole-body skeletal muscle mass divided by height squared (kg/m^2^).

After 15 min of seated rest, REE was measured in the seated position for approximately 5–10 min using the MedGem+ portable indirect calorimeter (Microlife Inc., Clearwater, FL, USA), and the data were analyzed using the dedicated MedGem Analyzer software (Microlife Inc., Clearwater, FL, USA). The device was operated according to the manufacturer’s standard procedure. No external calibration was performed by the investigators before measurement, and detailed information regarding the device’s internal calibration process was not displayed or recorded. Participants breathed through a single-use mouthpiece while wearing a nose clip. The MedGem+ measures oxygen consumption (VO_2_) but does not directly measure carbon dioxide production (VCO_2_). REE was calculated internally from VO_2_ using a modified Weir equation with a fixed respiratory quotient of 0.85: REE (kcal/day) = 6.931 × VO_2_ (mL/min). Measurement completion was determined by the device’s proprietary built-in algorithm. Because VCO_2_ was not measured and the detailed criteria of the proprietary algorithm were unavailable to the investigators, investigator-defined steady-state criteria based on both VO_2_ and VCO_2_ could not be applied. Measurements displaying an inadequate mouthpiece seal or another device-detected error were not accepted as completed measurements. One accepted MedGem+ measurement was obtained for each participant. A new single-use breathing attachment, including the mouthpiece and filter, was used for each measurement.

Conventional indirect calorimetry is generally regarded as the reference method for measuring REE. A systematic review of best-practice methods for RMR measurement reported that 10–20 min of rest before measurement is sufficient for the metabolic effects of routine activities of daily living to dissipate and that an abbreviated measurement of approximately 10 min can provide an accurate estimate when an appropriate steady state is achieved [17]. Accordingly, the 15 min seated rest and device-determined 5–10 min measurement period used in the present study were considered suitable for a pragmatic assessment of REE. In addition, Compher et al. compared MedGem with a Deltatrac metabolic cart under conditions closely resembling those used in the present study, including a 4 h fast, 15 min of rest, and abstention from exercise before measurement [18]. Although MedGem demonstrated acceptable repeatability, its individual-level agreement with Deltatrac was variable. Therefore, measured REE was treated as a portable indirect calorimetry comparator rather than as an error-free gold standard.

Because measurements were performed after at least 4 h without food intake and following 15 min of seated rest, the protocol did not meet the stricter conditions required for BMR measurement, particularly overnight fasting and supine rest. Accordingly, the measured physiological quantity was defined as REE rather than BMR. Although some of the evaluated equations were originally developed to estimate BMR, their equation-derived estimates were compared with REE measured by portable indirect calorimetry because these equations are commonly used to estimate resting energy requirements in clinical practice.

### 2.3. Predictive Equations

Four equation-derived estimates were calculated. The physiological quantity originally estimated by each method is identified below as BMR or REE according to the terminology used in the corresponding source. Body weight (BW) was expressed in kilograms, height in centimeters, age in years, and fat-free mass (FFM) in kilograms.

**Harris–Benedict equation [5]—BMR**

Men: Predicted BMR (kcal/day) = 66.47 + 13.75 × BW + 5.00 × height − 6.76 × age

Women: Predicted BMR (kcal/day) = 655.10 + 9.56 × BW + 1.85 × height − 4.68 × age

**Cunningham equation [6,7]—REE**

Predicted REE (kcal/day) = 370 + 21.6 × FFM

**Method based on the Dietary Reference Intakes for Japanese [8]—BMR**

Predicted BMR (kcal/day) = sex- and age-specific basal metabolic reference value (kcal/kg/day) × BW (kg)

**Ganpule equation [9,10]—BMR**

Men: Predicted BMR (kcal/day) = (0.1238 + 0.0481 × BW + 0.0234 × height − 0.0138 × age − 0.5473) × 1000/4.186

Women: Predicted BMR (kcal/day) = (0.1238 + 0.0481 × BW + 0.0234 × height − 0.0138 × age − 1.0946) × 1000/4.186

A comparison of participant backgrounds is shown in Table 1 and Supplementary Table S1.

### 2.4. Statistical Analysis

Resting energy expenditure (REE) measured by portable indirect calorimetry was used as the comparator measurement, and its agreement with REE predicted using the Harris–Benedict, Japanese Dietary Reference Intakes (DRIs), Cunningham, and Ganpule equations was evaluated. Statistical analyses were performed using R 4.5.3 (R Foundation for Statistical Computing, Vienna, Austria). The main packages used were dplyr (version 1.2.1) for data processing, ggplot2 (version 4.0.2) for figure creation, irr (version 0.85) for calculation of intraclass correlation coefficients (ICCs), and flextable and officer for table creation. The distributions of continuous variables were assessed using histograms and Q–Q plots, supplemented by Shapiro–Wilk tests. Means and standard deviations were retained as descriptive measures of magnitude and variability and were not interpreted as evidence of normality. For variables showing clear skewness, the median, interquartile range, and range were additionally reported.

The statistical measures were selected to evaluate distinct aspects of method performance. Spearman’s rank correlation coefficient was used to assess the monotonic association between measured and predicted REE; it was not interpreted as a measure of numerical agreement. Absolute-agreement reliability was evaluated using a two-way random-effects, single-measure intraclass correlation coefficient [ICC(2,1)] with its 95% confidence interval. ICC(2,1) is a sample-level reliability coefficient based on between-participant and error variance components and does not directly quantify the magnitude of prediction error for an individual participant. ICC values were interpreted according to Koo and Li as poor (<0.50), moderate (0.50–0.75), good (0.75–0.90), or excellent (>0.90), taking the 95% confidence interval into consideration [19].

Bland–Altman analysis was used to evaluate the magnitude and distribution of the differences between predicted and measured REE. The mean difference (bias) was used to describe the average direction of error, and the 95% limits of agreement (LoA) were used to describe the distribution of individual between-method differences. The mean bias, its 95% confidence interval, and the 95% LoA were calculated. Proportional bias was assessed by linear regression, with the between-method difference as the dependent variable and the mean of the two methods as the explanatory variable. The regression slope was used to determine whether the magnitude or direction of error varied according to the level of REE. The slope, its 95% confidence interval, and the corresponding *p* value were reported.

Percentage error was calculated as (predicted REE − measured REE)/measured REE × 100 and was used to describe the direction and relative magnitude of prediction error. Mean absolute percentage error was used to describe the average relative magnitude of error without cancellation of positive and negative values. The proportion of predictions within ±10% of measured REE was used as a descriptive measure of individual-level prediction accuracy and was reported with its 95% confidence interval calculated using the Wilson method. This ±10% threshold was not used as a prespecified criterion for establishing equivalence or interchangeability. Mean absolute error (MAE) was calculated to express the average absolute prediction error in kcal/day, whereas root mean square error (RMSE) was calculated as a complementary measure that gives greater weight to large individual errors.

The distributions of the paired differences and regression residuals were assessed separately using Q–Q plots and Shapiro–Wilk tests. Linearity and homoscedasticity were assessed by visual inspection of residual plots, and heteroscedasticity was additionally evaluated using the Breusch–Pagan test. Potentially influential observations were evaluated using studentized residuals and Cook’s distance. Because heteroscedasticity was observed in the proportional-bias regression models, HC3 heteroscedasticity-robust standard errors were used to calculate confidence intervals and *p* values for the regression slopes. As a sensitivity analysis, the proportional-bias regressions were repeated after excluding observations with Cook’s distance greater than 4/n, again using HC3 robust standard errors.

### 2.5. Multivariable Analysis of Relative Error

Because proportional bias was identified in the Bland–Altman analyses of the Harris–Benedict, Cunningham, and Ganpule equations, additional exploratory analyses were conducted to examine factors associated with relative prediction error for these three equations. For each equation, percentage residual error was calculated as follows:

percentage residual error = (predicted REE − measured REE)/measured REE × 100.

Percentage residual error was selected as the dependent variable because the raw between-method difference in kcal/day had already been evaluated using Bland–Altman analysis. Expressing the prediction error relative to measured REE permits comparison among participants with different absolute REE levels. For example, an error of 100 kcal/day represents 10% of a measured REE of 1000 kcal/day but only 5% of a measured REE of 2000 kcal/day. This relative-error scale is therefore useful when comparing participants with different body sizes and REE levels, including the different REE distributions observed between men and women. It is also consistent with the mean absolute percentage error and the proportion of predictions within ±10% of measured REE reported in this study. Thus, the multivariable analysis of percentage residual error was intended to complement, rather than duplicate, the Bland–Altman analysis of the raw between-method difference.

In the primary analysis, a multivariable linear regression model was constructed using Mean, sex, and BMI as explanatory variables. Mean was defined as (predicted REE + measured REE)/2 and corresponded to the x-axis of the Bland–Altman plot. Mean was included to describe whether relative prediction error varied according to the magnitude of the paired REE values. As a sensitivity analysis, BMI was replaced by age, resulting in a model that included Mean, sex, and age. Because both percentage residual error and Mean were derived from the same paired predicted and measured REE values, the coefficient for Mean was interpreted only as a descriptive component of the proportional-error structure, rather than as an independent participant characteristic or causal effect. This mathematical relationship is also inherent in conventional Bland–Altman proportional-bias analysis, in which the between-method difference is regressed on Mean.

Linearity and homoscedasticity were assessed by visual inspection of residual plots, residual normality was assessed using Q–Q plots, and potentially influential observations were examined using studentized residuals and Cook’s distance. Multicollinearity among the explanatory variables was assessed using variance inflation factors (VIFs), with values greater than 5 considered indicative of potentially important multicollinearity.

Given the limited sample size, the number of covariates was restricted to minimize overfitting, and covariates were selected a priori based on clinical relevance. SMI was not included because of its close relationship with energy expenditure and its potential collinearity with Mean, particularly for the Cunningham equation, which directly incorporates fat-free mass. All multivariable regression analyses were considered exploratory.

## 3. Results

### 3.1. Participant Characteristics

Thirty-six participants were recruited and completed all study measurements. All participants had complete data for measured REE, anthropometric variables, and the four predictive methods and were included in the analyses. No participant was excluded after enrollment. The participants comprised 16 men and 20 women. Age was right-skewed, with a mean of 28.92 ± 10.32 years, a median of 23.0 years (IQR, 22.0–32.25 years), and a range of 20–57 years. For descriptive purposes, 22 participants were aged 20–29 years, eight were aged 30–39 years, three were aged 40–49 years, and three were aged 50–59 years. Age remained a continuous variable in all statistical analyses. Mean BMI was 22.06 ± 3.11 kg/m^2^, with a range of 18.0–29.9 kg/m^2^, and mean body fat percentage was 25.45 ± 7.82%, with a range of 10.8–38.3%. Thus, the cohort consisted predominantly of young, healthy adults without extreme BMI values. SMI was 10.36 ± 0.98 kg/m^2^ in men and 8.14 ± 1.45 kg/m^2^ in women. Mean measured and predicted REE values ranged from approximately 1500 to 1700 kcal/day in men and from 1100 to 1200 kcal/day in women (Table 2).

### 3.2. Agreement of Four Predictive Methods with REE Measured by Portable Indirect Calorimetry

Spearman’s correlation coefficients between measured and predicted REE ranged from 0.750 to 0.800 and were statistically significant for all four equations (all *p* < 0.001). The corresponding ICC(2,1) values indicated generally moderate-to-good agreement, with ICC(2,1) values ranging from 0.736 to 0.766 (Table 3).

**Figure 1:**
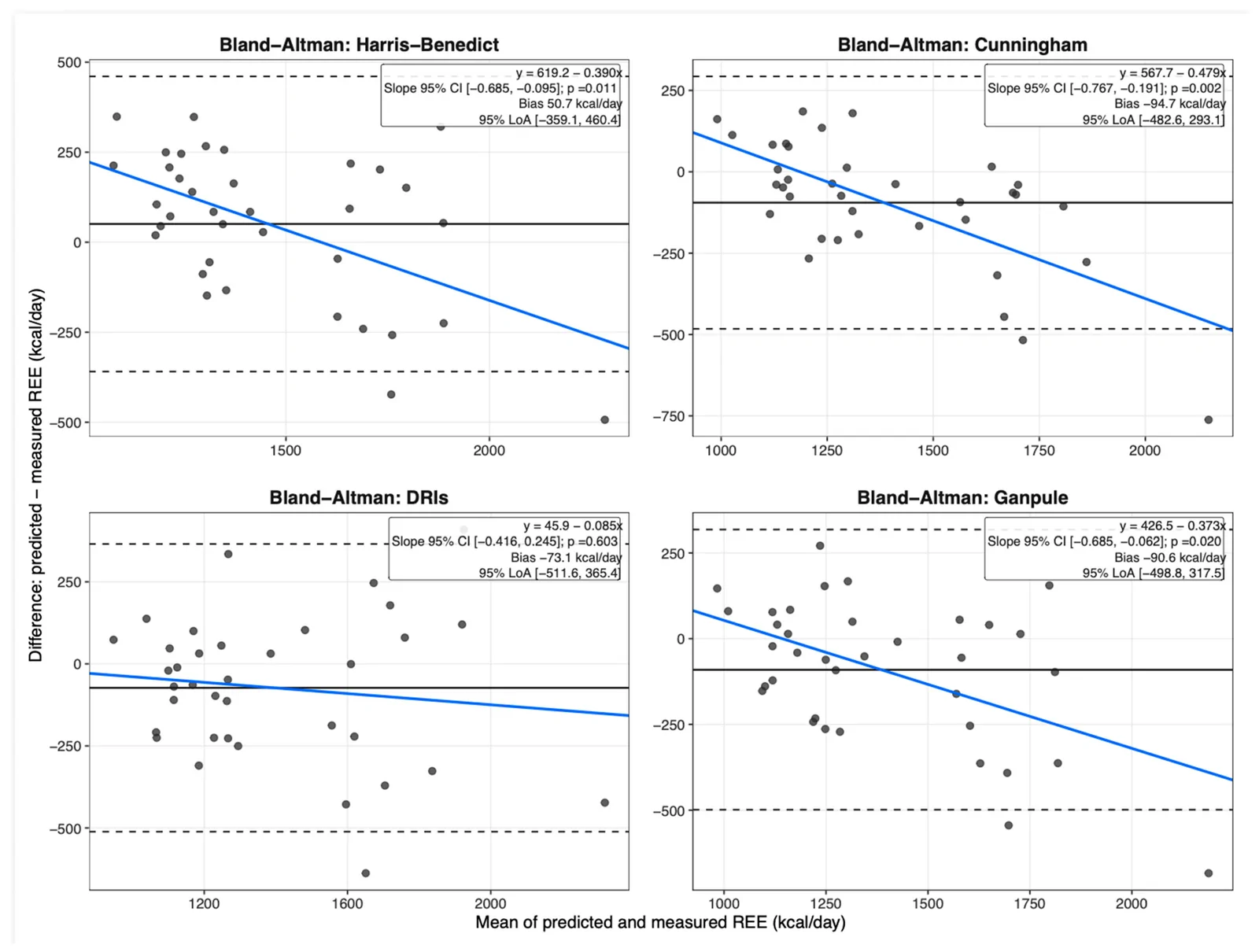
Bland–Altman analysis comparing values measured by portable indirect calorimetry and predicted values obtained from four REE methods. The x-axis represents the mean of the predicted and measured values, and the y-axis represents the difference between the predicted and measured values (predicted − measured). The solid black line represents the mean bias, the dashed lines represent the 95% limits of agreement (LoA), and the blue line represents the regression line used to assess proportional bias. Regression-slope confidence intervals and *p* values were calculated using HC3 heteroscedasticity-robust standard errors. The difference was defined as predicted REE minus measured REE, and the mean was calculated as (predicted REE + measured REE)/2. Abbreviations; REE: resting energy expenditure.

Mean bias was 50.7 kcal/day for the Harris–Benedict equation, whereas the DRIs, Cunningham, and Ganpule equations showed mean biases of −73.1, −94.7, and −90.6 kcal/day, respectively. The limits of agreement were wide for all four methods (Table 3 and Figure 1). Because heteroscedasticity was observed, these conventional limits of agreement were interpreted as descriptive overall summaries rather than as constant limits across the entire range of REE.

Visual inspection and Shapiro–Wilk testing did not indicate substantial departures from normality for the paired differences from the Harris–Benedict, DRIs, or Ganpule methods (*p* = 0.051, 0.961, and 0.119, respectively). In contrast, the Cunningham paired differences showed evidence of non-normality (*p* = 0.006). The residuals from the proportional-bias regression models did not show substantial departures from normality on Q–Q plots or Shapiro–Wilk testing (*p* = 0.336–0.994). However, heteroscedasticity was detected for all four methods. Using HC3 heteroscedasticity-robust standard errors, significant negative proportional bias was observed for the Harris–Benedict, Cunningham, and Ganpule equations, with slopes of −0.390 (95% CI, −0.685 to −0.095; *p* = 0.011), −0.479 (95% CI, −0.767 to −0.191; *p* = 0.002), and −0.373 (95% CI, −0.685 to −0.062; *p* = 0.020), respectively. The DRIs method showed no significant proportional bias [slope, −0.085 (95% CI, −0.416 to 0.245); *p* = 0.603].

No observation had an absolute studentized residual greater than 3. Cook’s distance greater than 4/n identified three potentially influential observations for the Harris–Benedict, DRIs, and Ganpule analyses and two for the Cunningham analysis. These observations were verified as valid measurements and retained in the primary analyses. In sensitivity analyses excluding these observations, negative proportional bias remained for the Harris–Benedict [slope, −0.327 (95% CI, −0.599 to −0.056); *p* = 0.020], Cunningham [−0.305 (95% CI, −0.498 to −0.113); *p* = 0.003], and Ganpule equations [−0.273 (95% CI, −0.517 to −0.029); *p* = 0.030], whereas the DRIs method remained without significant proportional bias [−0.030 (95% CI, −0.334 to 0.275); *p* = 0.845].

Mean percentage errors were 6.0%, −4.0%, −4.6%, and −4.5% for the Harris–Benedict, DRIs, Cunningham, and Ganpule methods, respectively. The corresponding mean absolute percentage errors were 12.9%, 12.0%, 10.0%, and 10.9%. The MAE values were 179.4, 181.2, 153.5, and 165.6 kcal/day for the Harris–Benedict, DRIs, Cunningham, and Ganpule methods, respectively. The corresponding RMSE values were 212.3, 232.4, 216.9, and 224.4 kcal/day. The proportions of predictions within ±10% of measured REE were 41.7% (95% CI, 27.1–57.8%) for Harris–Benedict, 50.0% (95% CI, 34.5–65.5%) for DRIs, 55.6% (95% CI, 39.6–70.5%) for Cunningham, and 52.8% (95% CI, 37.0–68.0%) for Ganpule (Table 3). Overall, although rank correlations and absolute agreement were broadly similar across the four methods, their patterns of systematic and proportional error differed. The DRIs, Cunningham, and Ganpule methods produced lower estimates on average, whereas significant negative proportional bias was observed for the Harris–Benedict, Cunningham, and Ganpule equations.

### 3.3. Factors Associated with Relative Prediction Error

For the exploratory multivariable models, residual plots and Q–Q plots did not indicate substantial violations of linearity, homoscedasticity, or residual normality. In the primary models including the mean, sex, and BMI, the mean of predicted and measured REE was inversely associated with percentage residual error for the Harris–Benedict, Cunningham, and Ganpule equations, with regression coefficients of −0.071 (95% CI, −0.096 to −0.046; *p* < 0.001), −0.055 (95% CI, −0.075 to −0.035; *p* < 0.001), and −0.064 (95% CI, −0.087 to −0.040; *p* < 0.001), respectively. Female sex was also associated with a more negative percentage residual error for all three equations: −15.64 percentage points (95% CI, −27.33 to −3.96; *p* = 0.010) for Harris–Benedict, −14.69 percentage points (95% CI, −24.22 to −5.16; *p* = 0.004) for Cunningham, and −18.17 percentage points (95% CI, −29.27 to −7.07; *p* = 0.002) for Ganpule. In contrast, BMI was positively associated with percentage residual error, with coefficients of 2.92 (95% CI, 1.35 to 4.48; *p* < 0.001), 1.36 (95% CI, 0.17 to 2.55; *p* = 0.027), and 2.76 (95% CI, 1.31 to 4.20; *p* < 0.001), respectively (Table 4). None of the VIF values exceeded the prespecified threshold of 5; the values ranged from 3.61 to 4.31 for the mean, from 2.77 to 2.96 for sex, and from 1.64 to 1.91 for BMI.

In the sensitivity models in which BMI was replaced by age, the mean of predicted and measured REE remained inversely associated with percentage residual error for all three equations. The corresponding coefficients were −0.044 (95% CI, −0.068 to −0.021; *p* < 0.001) for Harris–Benedict, −0.045 (95% CI, −0.063 to −0.026; *p* < 0.001) for Cunningham, and −0.037 (95% CI, −0.060 to −0.014; *p* = 0.003) for Ganpule. Female sex was associated with a more negative percentage residual error for the Cunningham equation [−14.47 percentage points (95% CI, −24.76 to −4.18); *p* = 0.007] and the Ganpule equation [−13.20 percentage points (95% CI, −26.39 to −0.02); *p* = 0.050]. Age was not significantly associated with percentage residual error for Harris–Benedict [−0.31 (95% CI, −0.72 to 0.10); *p* = 0.129], Cunningham [0.14 (95% CI, −0.16 to 0.45); *p* = 0.350], or Ganpule [−0.12 (95% CI, −0.51 to 0.27); *p* = 0.539] (Table 4).

Overall, in the primary models, higher mean and female sex were associated with greater underestimation, whereas higher BMI was associated with a shift toward overestimation. These associations should be interpreted cautiously because of the limited sample size, the exploratory nature of the analyses, and the equation-specific findings. In addition, because Mean was mathematically derived from both predicted and measured REE, which were also used to calculate percentage residual error, its association with percentage residual error may partly reflect mathematical coupling and should not be interpreted as an independent causal effect.

## 4. Discussion

In the present study, the participants’ age and body-size distributions broadly overlapped with the ranges represented in the source populations or data underlying the predictive methods, although differences remained in ethnicity, historical period, and metabolic measurement protocol. Thus, the equations were evaluated under relatively non-extreme conditions rather than extrapolated to individuals of advanced age or with extreme BMI values. Under these conditions, the relatively favorable group-level associations and absolute agreement may not be unexpected. Nevertheless, proportional bias was detected for three equations. The limits of agreement were wide in the present sample, although their precision was limited by the small sample size. Differences in ethnicity and metabolic measurement protocols should also be considered when interpreting equation-specific underestimation or overestimation.

In this study, rank associations were moderate to strong, whereas the ICC(2,1) point estimates indicated moderate-to-good absolute agreement according to the criteria of Koo and Li [19]. However, Spearman’s rho, ICC(2,1), the proportion of predictions within ±10%, and Bland–Altman analysis evaluate different aspects of method performance. Spearman’s rho reflects association but not numerical agreement. ICC(2,1) is a sample-level reliability coefficient for absolute agreement and is influenced by between-participant variability; it does not directly quantify the magnitude of prediction error for an individual participant. In contrast, the proportion of predictions within ±10% and the Bland–Altman limits of agreement more directly describe individual-level prediction error. Therefore, the moderate-to-good ICC values were not inconsistent with the relatively low proportions of predictions within ±10% and the wide limits of agreement. This distinction demonstrates that acceptable association and ICC values do not necessarily guarantee satisfactory individual-level prediction accuracy. The DRIs, Cunningham, and Ganpule methods produced lower estimates on average, although the 95% confidence interval for the mean bias included zero for the DRIs method. In the exploratory multivariable analyses, a higher mean and female sex were associated with more negative percentage residual error, whereas higher BMI was associated with a positive shift in percentage residual error. These equation-specific associations should be interpreted cautiously and require confirmation in larger independent samples.

First, the four prediction methods differed in their derivation populations and underlying measurement approaches. The Harris–Benedict equation was developed from an early 20th-century Western population, whereas the Cunningham equation was proposed based on a synthesis of studies examining the relationship between REE and fat-free mass. The Ganpule equation and the DRIs method were developed from Japanese data, although their underlying populations and measurement protocols differed [5,6,7,8,9,10]. This population dependency may have influenced the error structure. Ndahimana et al. compared multiple predictive equations and reported equation-specific differences in accuracy and population-dependent error patterns [20]. Frankenfield et al. also emphasized the limitations of applying predictive equations across different age and ethnic groups [11]. The expert panel recommended that acceptance of RMR estimates derived from predictive equations should be based on clinical judgment. Therefore, estimates obtained in populations that were poorly represented in the original studies should be interpreted cautiously. It is important to note that a strong association does not necessarily indicate the absence of bias or good individual-level agreement. The association of BMI with prediction error was broadly consistent with previous reports, whereas age was not significantly associated with percentage residual error in the present sample. Accordingly, particular caution is warranted when applying these equations to individuals with obesity or those who are underweight.

In clinical practice, these equations are widely used when indirect calorimetry is unavailable and are often applied to individuals whose characteristics extend beyond those represented in the original derivation studies. For example, Tabata et al. evaluated the Harris–Benedict and Ganpule equations in hospitalized patients with type 2 diabetes. However, because these equations were derived principally from healthy populations, their application to hospitalized patients with diabetes represents clinical extrapolation and addresses a different research question from that of the present study [21]. Rather et al. reported that prediction error increased in individuals with severe obesity and that the direction of error varied according to BMI [22]. Similarly, Jésus et al. reported that conventional prediction equations were inaccurate in individuals with either BMI < 16 kg/m^2^ or severe obesity [23]. In older adults, REE prediction equations have also shown substantial individual-level variability, with accuracy declining with advancing age [24]. Therefore, predicted REE should not be interpreted as a precise individual value but should be used with an understanding of the direction and magnitude of error associated with patient characteristics.

In actual nutritional management, it is common to calculate the required energy intake by incorporating activity factors and stress factors into the values obtained from estimation formulas. Since multiple estimation components are combined in this process, understanding the directionality of the underlying estimation errors may be clinically important. For example, even if the calculated energy intake appears sufficient, if the expected outcomes are not achieved, it may be necessary to consider the impact of systematic errors related to sex (female) and BMI.

In contrast to the Harris–Benedict, Cunningham, and Ganpule equations, the DRIs method did not show statistically significant proportional bias in the present sample. However, this finding should not be interpreted as evidence that the DRIs method is inherently less dependent on the magnitude of REE, because the confidence interval for the slope was wide and the sample size was limited.

This study has several limitations. First, this was a small pilot exploratory study involving 36 paired observations. Although this sample permitted estimation of mean bias, limits of agreement, and proportional-bias slopes, the limited sample size reduced the precision of these estimates, particularly the limits of agreement. The study was not designed to establish equivalence or interchangeability between equation-derived estimates and REE measured by portable indirect calorimetry. Similarly, the multivariable analyses were intended to identify potential error patterns rather than to provide confirmatory or predictive inference. The agreement estimates and exploratory associations should therefore be confirmed in a larger, prospectively designed independent study. In addition, the paired differences for the Cunningham equation showed evidence of non-normality, and heteroscedasticity was observed for all four methods. Therefore, the conventional limits of agreement should be regarded as descriptive overall summaries and may not represent constant agreement across the entire range of REE. Nevertheless, sensitivity analyses excluding potentially influential observations produced the same overall pattern of proportional bias.

Second, the relative homogeneity of the study population allowed us to examine whether individual-level prediction error persisted under comparatively non-extreme conditions, without the marked clinical heterogeneity associated with advanced age, extreme BMI, or acute and chronic disease. However, the study population was a convenience sample consisting exclusively of university staff members and undergraduate students and was predominantly composed of young adults. This substantially limits the external validity and generalizability of the magnitude and determinants of prediction error observed in this cohort, particularly to older adults, hospitalized patients, individuals with acute or chronic diseases, and those with extreme BMI values. Future studies should validate these error patterns in larger and more diverse clinical populations.

Third, this was a cross-sectional comparison study, and associations between estimation error and actual clinical outcomes following nutritional interventions were not evaluated. Therefore, the present findings should be considered exploratory and hypothesis-generating. Future large-scale validation studies involving more diverse Japanese patient populations are therefore needed to confirm the reproducibility and clinical applicability of these findings, as well as to clarify their relationship with nutritional management outcomes.

Fourth, the measurements in the present study were performed under resting rather than strict basal conditions. Measurement of BMR generally requires an overnight fast of approximately 12 h, whereas participants in the present study were examined at least 4 h after their last meal; therefore, the measured values should be interpreted as REE rather than BMR. Although REE and BMR are closely related physiological measures and REE is generally reported to be approximately 5–10% higher than BMR, residual diet-induced thermogenesis cannot be completely excluded under the present protocol. Nevertheless, the relatively modest expected difference between REE and BMR is unlikely to fully explain the wide individual-level variation and proportional bias observed in this study.

Fifth, the portable indirect calorimeter used in this study measured VO_2_ but not VCO_2_ and calculated REE using a modified Weir equation with a fixed RQ of 0.85. If the actual RQ was lower or higher than 0.85, measured REE could have been overestimated or underestimated, respectively. Thus, the fixed-RQ assumption may have affected the mean bias, limits of agreement, and proportional-bias slopes observed in the Bland–Altman analyses. Furthermore, unlike a full metabolic cart using a ventilated hood, the device did not permit the application of investigator-defined steady-state criteria based on both VO_2_ and VCO_2_. Previous comparisons of MedGem with full metabolic carts have also reported variable systematic bias and wide individual-level limits of agreement [18,25]. Therefore, the disagreement observed in the present study should not be attributed exclusively to the prediction equations but may also partly reflect measurement error associated with the portable indirect calorimetry protocol. Although the portability and operational simplicity of the MedGem+ may facilitate its clinical use, measured REE should be interpreted as a portable indirect calorimetry measurement rather than as an error-free gold-standard value.

Sixth, several potential determinants of REE were not comprehensively assessed or standardized. Although women using oral contraceptives and those with evident menstrual disorders were excluded, menstrual-cycle phase was not recorded or controlled for. Therefore, menstrual phase-related fluctuations in REE may have contributed to the observed individual-level variability. Body composition was assessed using bioelectrical impedance analysis; however, detailed metabolic health status and organ- or tissue-specific body composition were not available. Smoking status, habitual and recent physical activity, caffeine and alcohol intake, and dietary intake and composition were not systematically assessed. These unmeasured factors may have contributed to the observed individual-level variability in REE.

Seventh, the exploratory multivariable analyses have several methodological limitations. Percentage residual error was selected to permit comparison of prediction error across participants with different absolute REE levels and to complement the Bland–Altman analysis of the raw between-method difference. For example, the same absolute error of 100 kcal/day corresponds to 10% when measured REE is 1000 kcal/day but to 5% when measured REE is 2000 kcal/day. This relative scale also allows interpretation within the same framework as the mean absolute percentage error and the proportion of predictions within ±10% of measured REE. Nevertheless, Mean was mathematically derived from the same predicted and measured REE values used to calculate percentage residual error. This mathematical relationship is also inherent in conventional Bland–Altman proportional-bias analysis, in which the between-method difference is regressed on Mean. Therefore, the coefficient for Mean was interpreted only as a descriptive component of the proportional-error structure and not as an independent participant characteristic or causal effect.

After accounting for Mean, sex and BMI remained associated with percentage residual error in the primary models. These coefficients represent conditional, equation-specific associations rather than causal effects. The associations were not fully consistent across the models in which BMI was replaced by age and should therefore be considered hypothesis-generating and confirmed in larger independent samples.

## 5. Conclusions

In this pilot exploratory study, the four predictive methods showed moderate-to-strong rank associations with REE measured by portable indirect calorimetry. Nevertheless, proportional bias was detected for the Harris–Benedict, Cunningham, and Ganpule equations in participants with relatively non-extreme age and body-size distributions. Exploratory analyses suggested equation-specific associations of prediction error with sex and BMI. The limits of agreement were estimated descriptively, but their precision was limited by the small sample size. Prediction equations should therefore be used with an understanding that the direction of error may vary according to the equation and participant characteristics. Future studies should include larger and more diverse populations, particularly older adults and individuals with acute or chronic diseases, to evaluate the generalizability and clinical applicability of these findings.

## Figures and Tables

**Table 1:** Key characteristics of the source data underlying the four predictive methods and the present study.

Characteristic	Harris–Benedict	DRIs	Cunningham	Ganpule	Present Study
Original outcome	BMR	BMR	REE	BMR	REE
Source population	US adults	Japanese reference data	Reanalysis/synthesis of body-composition data	Japanese adults	Japanese adults
Sample size	239	—	223	137	36
Age range, years	16–63	Adult age categories	16–63	20–79	20–57
Measurement basis	Respiratory calorimetry	Basal metabolic reference values	FFM-based model	Indirect calorimetry/metabolic chamber	Portable indirect calorimetry

Abbreviations: BMR, basal metabolic rate; REE, resting energy expenditure; DRIs, Dietary Reference Intakes for Japanese; FFM, fat-free mass.

**Table 2:** Participant characteristics.

	*Total* (*n* = 36)	*Male* (*n* = 16)	*Female* (*n* = 20)
Age, mean ± SD, years	28.92 ± 10.32	26.44 ± 7.82	30.90 ± 11.78
Age, median [IQR], years	23.0 [22.0–32.25]	23.0 [21.75–30.25]	23.0 [22.0–39.5]
Height (cm)	164.30 ± 8.63	171.22 ± 5.86	158.77 ± 6.13
BW (kg)	60.15 ± 13.28	69.46 ± 13.54	52.70 ± 6.98
BMI (kg/m^2^)	22.06 ± 3.11	23.58 ± 3.79	20.84 ± 1.72
SMI(kg/m^2^)	8.96 ± 1.47	10.36 ± 0.98	8.14 ± 1.45
Body fat percentage (%)	25.45 ± 7.82	20.66 ± 8.50	29.29 ± 4.57
Measured REE (kcal/day)	1431.11 ± 349.69	1709.38 ± 330.99	1208.50 ± 146.30
Harris (kcal/day)	1481.77 ± 244.45	1698.96 ± 199.63	1308.02 ± 84.32
DRIs (kcal/day)	1358.04 ± 324.08	1614.10 ± 300.05	1153.19 ± 151.69
Cunningham (kcal/day)	1336.36 ± 222.37	1542.85 ± 156.17	1171.17 ± 84.86
Ganpule (kcal/day)	1340.48 ± 248.41	1566.97 ± 173.81	1159.28 ± 109.85

Skeletal muscle mass index (SMI) was calculated as whole-body skeletal muscle mass divided by height squared (kg/m^2^). Data are presented as mean ± SD unless otherwise indicated. Median and IQR are additionally reported for age because of its right-skewed distribution. Abbreviation: IQR: Interquartile range; BW: body weight; BMI: body mass index; REE: Resting energy expenditure; DRIs: Dietary reference intakes.

**Table 3:** Agreement and prediction error of four predictive equations compared with portable indirect calorimetry (n = 36).

Measure	Harris–Benedict	DRIs	Cunningham	Ganpule
Spearman’s ρ	0.760, *p* < 0.001	0.750, *p* < 0.001	0.800, *p* < 0.001	0.764, *p* < 0.001
ICC (2,1), absolute agreement	0.754 (0.571–0.866)	0.766 (0.584–0.874)	0.738 (0.502–0.865)	0.736 (0.514–0.861)
Mean bias (95% CI) [95% LoA], kcal/day	50.7 (−20.1 to 121.4) [−359.1, 460.4]	−73.1 (−148.8 to 2.6) [−511.6, 365.4]	−94.7 (−161.7 to −27.8) [−482.6, 293.1]	−90.6 (−161.1 to −20.2) [−498.8, 317.5]
Proportional-bias slope (95% CI), *p* value	−0.390 (−0.685 to −0.095), *p* = 0.011	−0.085 (−0.416 to 0.245), *p* = 0.603	−0.479 (−0.767 to −0.191), *p* = 0.002	−0.373 (−0.685 to −0.062), *p* = 0.020
Percentage error, mean ± SD, %	6.0 ± 14.5	−4.0 ± 14.2	−4.6 ± 11.7	−4.5 ± 12.7
Absolute percentage error, mean ± SD, %	12.9 ± 8.6	12.0 ± 8.3	10.0 ± 7.5	10.9 ± 7.8
Predictions within ±10% error, %	41.7% (27.1–57.8)	50.0% (34.5–65.5)	55.6% (39.6–70.5)	52.8% (37.0–68.0)
Mean absolute error, kcal/day	179.4	181.2	153.5	165.6
Root mean square error, kcal/day	212.3	232.4	216.9	224.4

Values in parentheses are 95% confidence intervals. Mean bias was calculated as predicted REE minus measured REE. Proportional bias was assessed by linear regression of the difference between predicted and measured REE on the mean of the two values. Percentage error was calculated as (predicted REE − measured REE)/measured REE × 100. Absolute percentage error was calculated as |predicted REE − measured REE|/measured REE × 100. The slope represents the change in the prediction difference associated with a 1 kcal/day increase in the mean. Abbreviations: DRIs, Dietary Reference Intakes for Japanese; ICC, intraclass correlation coefficient; LoA, limits of agreement; REE, resting energy expenditure; SD, standard deviation; CI, confidence interval.

**Table 4:** Exploratory multivariable analyses of percentage residual error.

Variable	Harris–Benedict	Cunningham	Ganpule
**Model 1: Mean, sex, and BMI**			
Mean	−0.071 (−0.096 to −0.046); *p* < 0.001	−0.055 (−0.075 to −0.035); *p* < 0.001	−0.064 (−0.087 to −0.040); *p* < 0.001
Female sex	−15.64 (−27.33 to −3.96); *p* = 0.010	−14.69 (−24.22 to −5.16); *p* = 0.004	−18.17 (−29.27 to −7.07); *p* = 0.002
BMI	2.92 (1.35 to 4.48); *p* < 0.001	1.36 (0.17 to 2.55); *p* = 0.027	2.76 (1.31 to 4.20); *p* < 0.001
**Model 2: Mean, sex, and age**			
Mean	−0.044 (−0.068 to −0.021); *p* < 0.001	−0.045 (−0.063 to −0.026); *p* < 0.001	−0.037 (−0.060 to −0.014); *p* = 0.003
Female sex	−10.40 (−23.75 to 2.94); *p* = 0.122	−14.47 (−24.76 to −4.18); *p* = 0.007	−13.20 (−26.39 to −0.02); *p* = 0.050
Age	−0.31 (−0.72 to 0.10); *p* = 0.129	0.14 (−0.16 to 0.45); *p* = 0.350	−0.12 (−0.51 to 0.27); *p* = 0.537

Values are regression coefficients (95% confidence intervals); *p* values. Percentage residual error was calculated as (predicted REE − measured REE)/measured REE × 100. Mean was defined as (predicted REE + measured REE)/2. Male sex was the reference category. Model 1 included Mean, sex, and BMI; Model 2 included Mean, sex, and age.

## Data Availability

The datasets presented in this article are not readily available because the data are part of an ongoing study. Requests to access the datasets should be directed to the correspondence author.

## Supplementary Materials

The following supporting information can be downloaded at: https://www.mdpi.com/article/10.3390/nu18162743/s1, Table S1: Participants’ Baseline Characteristics and Formulas.
