# Supplementary material for: Bias Characterization of Resting Energy Expenditure Prediction Equations in Japanese Subjects: A Pilot Exploratory Study
Source: Nutrients. 2026 Aug 21;18(16):2743. doi: 10.3390/nu18162743 (PMC13516483; doi:10.3390/nu18162743)
Supplement: Supplementary file 1 [file nutrients-18-02743-s001.zip › nutrients-4430391-supplementary.pdf]

Supplementary Table S1. Participants' Baseline Characteristics & Formulas

**Item / Parameter**	**Harris-Benedict Equation (1919)**	**Dietary Reference Intakes for Japanese (2025)**	**Cunningham Equation (1991)**	**Ganpule Equation (2007)**	**Our study**
**Reference**	**5**	**8**	**6, 7**	**9**	**ND**
Abbreviation in this study	Harris	DRIs	Cunningham	Ganpule	Our study
Country / Ethnicity	USA / Predominantly Caucasian	Japan / Japanese	USA / Predominantly Caucasian	Japan / Japanese	Japan / Japanese
Measurement Method	Early closed-circuit respiratory apparatus	Whole-body indirect calorimetry (Chamber, etc.)	Reanalysis of HB (1919) raw data	Human Metabolic Chamber	
Total Sample Size	239	341 (Core data pool for adults)	223	137	36
Sex Distribution	Male: 136 / Female: 103	Male: 169 / Female: 172	Male: 120 / Female: 103	Male: 71 / Female: 66	Male:16/Female:20
Age Range (Years)	16 – 63	18 – 70+ (All adult age groups)	16 – 63 (Same as HB data)	20 – 79	20-57
Mean Age (Males)	Approx. 27.0 ± 8.8 y	Categorized by age groups	Approx. 27 y	41.5 ± 15.5 y	26.44±7.82 y
Mean Age (Females)	Approx. 31.1 ± 10.1 y	Categorized by age groups	Approx. 31.1 y	45.8 ± 16.0 y	30.90±11.78 y
Mean Height (Males)	Approx. 174.0 cm	Reference Height (e.g., 30-49y): 170.0 cm	Slightly lower than HB	169.1 ± 5.8 cm	171.22±5.86 cm
Mean Height (Females)	Approx. 162.0 cm	Reference Height (e.g., 30-49y): 158.0 cm	Approx. 162.0 cm	155.6 ± 5.4 cm	158.77±6.13 cm
Mean Weight (Males)	Approx. 61.1 kg	Reference Weight (e.g., 30-49y): 68.5 kg	Lower than HB (Athletes excluded)	67.2 ± 11.1 kg	69.46±13.54 kg
Mean Weight (Females)	Approx. 56.5 kg	Reference Weight (e.g., 30-49y): 53.1 kg	Approx. 56.5 kg	53.6 ± 7.9 kg	52.70±6.98 kg

Mean BMI (Males)	Approx. 20.2 (Lean)	Approx. 23.7 (Modern Japanese Mean)	—	Approx. 23.5	23.58±3.79
Mean BMI (Females)	Approx. 21.5 (Normal)	Approx. 21.3 (Normal)	Approx. 21.5	Approx. 22.1	20.84±1.72
